# Supplementary material for: Chromothripsis during telomere crisis is independent of NHEJ, and consistent with a replicative origin
Source: Genome Res. 2019 May;29(5):737–49. doi: 10.1101/gr.240705.118 (PMC6499312; doi:10.1101/gr.240705.118)
Supplement: Supplemental Material [file supp_gr.240705.118_Supplemental_file_1.zip › contigs/annotated_contigs/DB79/contig.4.DB79_length_570_mean_cov_22.1807017544.docx]

**DB79_length_570_mean_cov_22.1807017544**

```
TCCGCGTGGTCCCAGCTCCACCGCGGGCCGAGGCCGGTGGAGGTCGCGGGCGCGCGAACGGGAATCGACGCCCTCGC|GT|TTACAGAT
 >chrUn_gl000220:119881-119960 - E=8e-27                                      >chr3:142
CACAAGGTCACTGAAATAAATCACTATGTGAAAAAAAACAGCTATGAAATAGTTGAAGAATTTTCCTTCTAGTTTAAAGGAAAACCTGG
375374-142375582 - E=2e-113
AAAATATTTTCATCTATATGTCTAGCAATCTCCTTATTCAACAACATTTCACAACAGGGAGCTGTATCATTCATTTTGGAAAAAGTAAG

TATTCATATTCCA|TATCAAC|AAACATTTTTAAATTTCCCAGCAGTTAACGAATAGTG|A|GGGCAAAAGCTCGCTTGATCTTGATTT
              >chr1:212901171-212901216 - E=2e-15         >chr1:91852789-91853037 - E
TCAGTACGAATACAGACCGTGAAAGCGGGGCCTCACGATCCTTCTGACCTTTTGGGTTTTAAGCAGGAGGTGTCAGAAAAGTTACCACA
=1e-137
GGGATAACTGGCTTGTGGCGGCCAAGCGTTCATAGCGACGTCGCTTTTTGATCCTTCGATGTCGGCTCTTCCTATCATTGTGAAGCAGA

ATTCACCAAGCGTTGGATTGTTCACCCACTAATAGGGAACGT
```
